# Mechanism of action of paclitaxel for treating glioblastoma based on single-cell RNA sequencing data and network pharmacology

**DOI:** 10.3389/fphar.2022.1076958

**Published:** 2022-11-21

**Authors:** Jianglong Lu, Fanjie Xu, Changjun Rao, Chaodong Shen, Jinghao Jin, Zhangzhang Zhu, Chengde Wang, Qun Li

**Affiliations:** Department of Neurosurgery First Affiliated Hospital of Wenzhou Medical University, Wenzhou, China

**Keywords:** glioblastoma, paclitaxel, single-cell RNA sequencing, bioinformatics, network pharmacology

## Abstract

Paclitaxel is an herbal active ingredient used in clinical practice that shows anti-tumor effects. However, its biological activity, mechanism, and cancer cell-killing effects remain unknown. Information on the chemical gene interactions of paclitaxel was obtained from the Comparative Toxicogenomics Database, SwishTargetPrediction, Binding DB, and TargetNet databases. Gene expression data were obtained from the GSE4290 dataset. Differential gene analysis, Kyoto Encyclopedia of Genes and Genomes, and Gene Ontology analyses were performed. Gene set enrichment analysis was performed to evaluate disease pathway activation; weighted gene co-expression network analysis with diff analysis was used to identify disease-associated genes, analyze differential genes, and identify drug targets *via* protein-protein interactions. The Molecular Complex Detection (MCODE) analysis of critical subgroup networks was conducted to identify essential genes affected by paclitaxel, assess crucial cluster gene expression differences in glioma *versus* standard samples, and perform receiver operator characteristic mapping. To evaluate the pharmacological targets and signaling pathways of paclitaxel in glioblastoma, the single-cell GSE148196 dataset was acquired from the Gene Expression Omnibus database and preprocessed using Seurat software. Based on the single-cell RNA-sequencing dataset, 24 cell clusters were identified, along with marker genes for the two different cell types in each cluster. Correlation analysis revealed that the mechanism of paclitaxel treatment involves effects on neurons. Paclitaxel may affect glioblastoma by improving glucose metabolism and processes involved in modulating immune function in the body.

## 1 Introduction

The most common primary malignant brain tumors of the central nervous system are gliomas, which originate from neuroectodermal cells (Jiang et al., 2011a; Wang et al., 2016) and are responsible for 74.6% of malignant tumors and 24.7% of initial brain tumors (Mat Zin and Zulkarnain, 2019). Gliomas are characterized by rapid growth, aggression, relapse after surgery, and a high death rate (Liu et al., 2012). Surgery, chemotherapy, radiation, and other methods are the main treatment options for glioblastoma (Yang et al., 2013). Treatments for glioblastoma have advanced in recent years through the development of chemotherapeutic medicines. Chemical drugs improve outcomes following surgery or radiation therapy and prolong the survival time and tumor-free survival time (Chen et al., 2017). However, the targets of chemotherapeutic drugs are unclear, the drugs do not easily cross the blood-brain barrier, and their effects are insufficient; additionally, effective drugs do not concentrate at the lesion site and do not remain at this site long-term (Johnson and Phillips, 1996). These drugs also show low bioavailability (Talibi et al., 2014). Thus, new treatment options for glioblastoma are needed (Bush et al., 2017).

The anticancer drug paclitaxel is extracted from the bark of the yew tree and targets microtubule proteins (Bastiancich et al., 2019). Paclitaxel accelerates the formation of microtubules from microtubule dimers and prevents their separation, which induces abnormal mechanical reorganization of the microtubules and inhibits normal cell division. This drug also inhibits the effects of other factors on the microtubule system and, together with the stable binding of microtubule proteins, eventually induces apoptosis (Liu et al., 2017a). In clinical applications, paclitaxel has shown good efficacy in treating non-small cell lung cancer (Hoang et al., 2012), breast cancer (Manhas et al., 2022), gastric cancer (Tu et al., 2022), nasopharyngeal cancer (Xia et al., 2022), ovarian cancer (Kong et al., 2023)and cervical cancer (Yasunaga et al., 2022), particularly for drug-resistant tumors (Song et al., 2018; Kawiak et al., 2019). Although the therapeutic efficacy of paclitaxel in glioma has been confirmed, its therapeutic mechanism remains unclear.

In addition, the activity of paclitaxel against brain tumors was disappointing in phase II experiments due to the presence of the blood-tumor barrier (BTB) and/or blood-brain barrier (BBB) (Zhang et al., 2012). In recent years, more and more studies have been devoted to the combined administration to break through the blood-brain barrier and act precisely on gliomas, and p-glycoprotein has been confirmed to be an important obstacle to preventing paclitaxel from entering the brain through studies of paclitaxel crossing the blood-brain barrier *in vitro* and *in vivo* (Fellner et al., 2002; Zhang et al., 2012; Li et al., 2016). One study showed angiopep-2 modified cationic liposomes for effective co-delivery of therapeutic genes encoding human tumor necrosis factor-related apoptosis-inducing ligand (pEGFP-hTRAIL) and paclitaxel to gliomas (Sun et al., 2012). Local delivery of brain-penetrating nanoparticles significantly improved the efficacy of paclitaxel for malignant gliomas and substantially delayed tumor growth (Nance et al., 2014). These studies and methods provide great help for paclitaxel to break through the blood-brain barrier and act as a precise drug-targeted therapy, and also make our study meaningful. This study was conducted to evaluate the specific effects of paclitaxel on glioblastoma and provide a new approach for treating this disease in clinical settings.

In this study, we investigated the mechanism of action of paclitaxel in glioblastoma therapy by using network, pharmacology, and genetics analyses. We determined the crucial role of immune function regulation in the prognosis of patients with glioblastoma. Analysis of transcription data from the Gene Expression Omnibus (GEO) database and corresponding clinical information revealed differentially expressed genes (DEGs). We also explored the correlations between drug- and disease-acting genes and levels of immune function activation, constructed a glioblastoma prediction model, and identified several different genes associated with immune activation as potential biomarkers. The findings were validated using the GEO single-cell dataset. Our findings revealed a crucial role for immunomodulation in treating glioblastoma with paclitaxel, which may act on neuronal cells and improve processes such as glucose metabolism to regulate the body’s immune function.

## 2 Materials and methods

**FIGURE 1:**
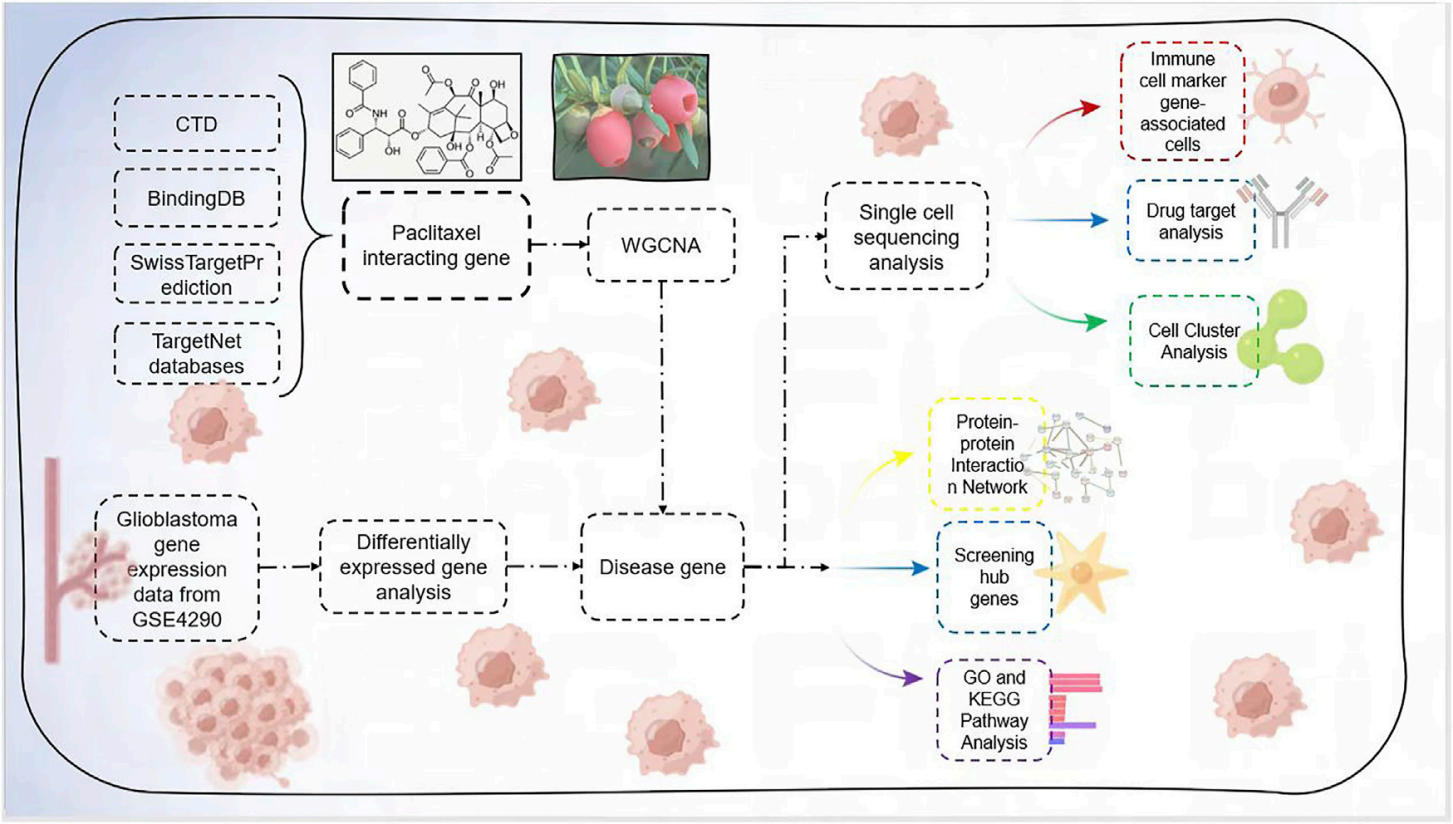
Flowchart.

Flowchart was shown in Figure 1.

### 2.1 Identification of targets of paclitaxel

The SwissTargetPrediction database (https://www.swisstargetprediction.ch/) was used to query paclitaxel for its targets and associated target genes. The Comparative Toxicogenomics Database (CTD; https://ctdbase.org/), BindingDB (http://bindingdb.org/bind/index.jsp), and TargetNet database (https://targetnet.scbdd.com/) were also used to identify potential target genes. Additionally, we used the UniProt database (https://www.uniprot.org/uploadlists/) to query genes corresponding to potential target proteins to screen for active ingredients. Our results were used to locate paclitaxel lactones through Excel searching and sorting. Gene Ontology (GO) was used for functional annotation. The Database for Annotation, Visualization, and Integrated Discovery (DAVID) (https://david.ncifcrf.gov/) was employed for functional annotation, Kyoto Encyclopedia of Genes and Genomes (KEGG) pathway analysis, and Disease Ontology (DO) functional annotation of the target genes. DAVID integrates biological information and statistical tools to help researchers identify gene and protein material. The bioinformatics tool GO analyzes and classifies biological processes into genes, with molecular functions, biological processes, and cells as the three GO components. Molecular data obtained using high-volume experimental techniques can be utilized to investigate signaling pathways, including numerous protein interactions and activities that regulate cellular function and metabolic activity. The ggplot2 tool in R was used to visualize the data, and an adjusted *p* < 0.05 was utilized for barrier testing.

### 2.2 Identification of DEGs in glioblastoma

77 glioblastoma samples and 23 healthy controls comprised the GSE4290 microarray dataset downloaded from the GEO database (http://www.ncbi.nlm.nih.gov/geo). To obtain a gene expression matrix for the samples, it was first normalized and integrated. The genomes of glioblastoma samples and healthy controls were analyzed using the R package “limma (version 3.5.1)". *p*-values were adjusted using the Benjamin–Hochberg method. The segmentation criteria were modified to |[log2 fold-change]| >1 and *p* < 0.05. Using the R packages “ggplot2 (version 3.3.2)” and “heatmap (version 0.3.2),” all genes were displayed in a volcano plot. Heatmap (version 0.7.7) was used to show the top 20 DEGs (Ito and Murphy, 2013). Ridge plots were designed, and the defined genomes were analyzed using gene set enrichment analysis (GSEA) to identify significant differences between the two characteristics (Subramanian et al., 2007). The biological pathways and processes involved in the pathogenesis of module membership (MM) were predicted using GSEA (version 3.0, http://www.gsea-msigdb.org/gsea/index.jsp). Hub gene expression values were employed as phenotype files to calculate Pearson correlation coefficients, and the KEGG pathway gene set was used as an enrichment background. The above gene sets were used as background genes for enrichment analysis, and the correlation coefficients of each hub gene with other genes were sorted in descending order as scan sequences. Analyses were performed using the following settings: false discovery rate <0.25, nominal *p*-value < 0.05, |normalized enrichment score| > 1.

### 2.3 Weighted gene co-expression network analysis of GEO

In the weighted gene co-expression network analysis (WGCNA) package of the R software, 5,000 genes with the highest average expression were selected to construct a weighted gene co-expression network using expression as a screening condition. The screening threshold was set to convert the paired correlation matrix into a neighborhood correlation matrix to ensure that the scale-free network calculated the paired Pearson correlation coefficients between all genes individually. The minimum number of genes per gene module was set to 30 using the dynamic hybrid shear tree algorithm criterion, and eigenvector values were calculated for each module. The modules were analyzed by clustering, and close modules were combined into a new module. The WGCNA algorithm calculates the module feature correlation to determine the correlation between module genes and disease subgroup phenotypes, and the heat map reflects the strength of the correlation. Individual modules were considered as significantly correlated with the phenotype when *p* < 0.05. The module showing the highest correlation coefficient with glioblastoma was selected as the key module. Pearson’s correlation coefficients were calculated for each co-expression module with gene identity values to screen for key genes. Genes with module membership (MM) > 0.8 and gene importance (GS) > 0.65 were selected as key genes. Differential genes were intersected with WGCNA as disease-related genes and imported into DAVID 6.8 for GO and KEGG pathway enrichment analyses. Pathway enrichment analysis was performed to validate the significant gene functional categories (*p* < 0.05).

### 2.4 Generation of protein-protein interaction networks

Protein-protein interactions (PPIs) were investigated using the cross-targets identified in STRING (version 10.5, https://string-db.org/). The network nodes and edges depict protein and high-binding conversations, respectively. Cytoscape software was used to create and visualize the PPI interaction networks (version 3.6.0). The Molecular Complex Detection (MCODE) algorithm detects dense regions of tightly linked protein or PPI networks and is used to screen for critical subnetworks that contribute to glioblastoma development, derive essential subpopulation genes, and perform GO enrichment analysis.

### 2.5 Differential expression of crucial subpopulation genes in glioblastoma and normal tissues

Differential expression analysis of crucial subpopulation genes was performed on the GEO dataset using statistical software R4.1.3 (The R Project for Statistical Computing, Vienna, Austria). Differential expression of crucial subpopulation genes between disease and control groups was explored under screening conditions of *p*-value < 0.05 and |[(log2 fold-change)]| > 1 and visualized as heat maps in R language. Data from GSE4290 were used to construct a disease control model validation set to assess the association of critical genes with glioblastoma in R language software (Robin et al., 2011). Receiver operating characteristic (ROC) curves were plotted, and screened core genes were evaluated by calculating the area under the ROC curve.

### 2.6 Single-cell RNA sequencing data analysis and identification of glioblastoma-associated genes

The original expression profile dataset (GSE148196) used for analysis was screened using the GEO public database. The dataset consisted of biopsies from four patients with active glioblastoma. Tissues were extracted and then analyzed using expression profiling microarrays on the Illumina NextSeq assay platform. The raw dataset was preprocessed using the Seurat R package to ensure the quality of the results. The total number of molecules within the cell (nCount RNA) and genes detected in each cell (nFeature RNA) were determined, and the number of genes was compared to the number of reads obtained from sequencing of each cell. Widespread mitochondrial genomic contamination in low-quality or dead cells was assessed by calculating the number of reads paired with the mitochondrial genome using a percentage feature set function. Cells were clustered based on the filtered principal components and visually classified using the unified manifold approximation and projection dimensionality reduction technique. Immune cell marker genes with adjusted *p*-values < 0.05 were screened. Immune cell marker genes were retrieved using the PanglaoDB database and intersected with the corresponding genes for each class group to identify the class group of the immune cells. The results revealed the potential targets of paclitaxel in glioblastoma.

### 2.7 Statistical analysis

A two-sided *p*-value of 0.05 was considered to indicate statistically significant results. Rstudio (www.r-project.org; version 4.2.1) was used to sort and observe the data (Packages: limma, edgeR, ggplot2, survminer, survival, RMS, randomForest, pROC, glmnet, heatmap, timeROC, *via* storyline, complot, ConsensusClusterPlus, forest plot, survival rock, beeswarm, edgeR, “TxDb.Hsapiens.UCSC.hg38,” “known gene,” “cluster profile,” “org.Hs.eg.DB,” “karyoploteR,” “GSVA,” “GSEABase,” “stringr,” “GEOquery,” “dplyr,” “ComplexHeatmap,” and “RColorBrewer”).

## 3 Results

### 3.1 Target genes of paclitaxel

**FIGURE 2:**
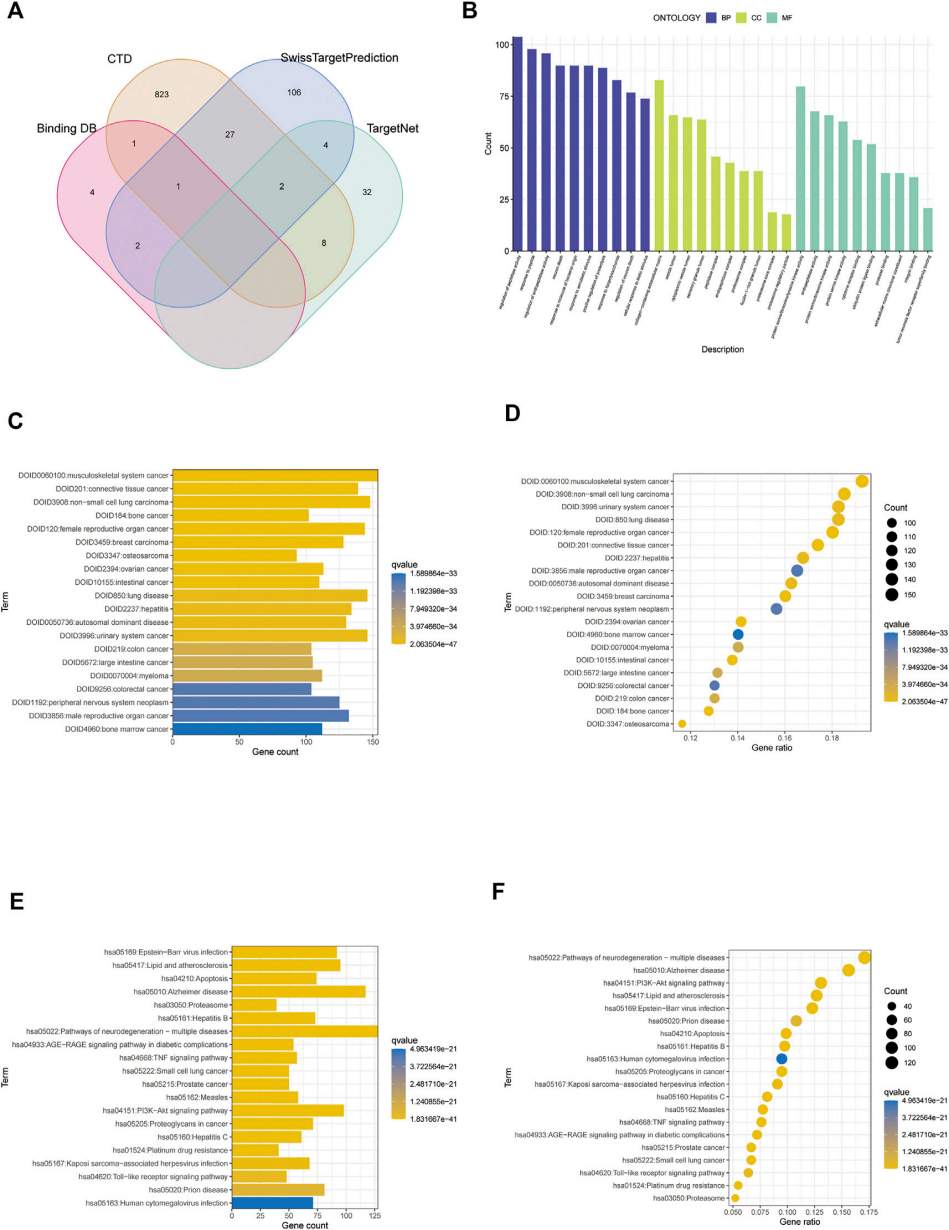
Screening analysis of paclitaxel targets. **(A)** Venn diagram of paclitaxel in the four databases. **(B)** Gene Ontology (GO) enrichment analysis of paclitaxel targets. **(C,D)** DO enrichment analysis of paclitaxel targets. **(E,F)** Kyoto Encyclopedia of Genes and Genomes (KEGG) enrichment analysis of paclitaxel targets.

Using the SwissTargetPrediction, CTD, BindingDB, and TargetNet databases, we identified and retrieved 1,010 target genes associated with paclitaxel lactone (Figure 2A; Supplementary Table S1). We performed GO and DO functional enrichment and KEGG pathway enrichment analyses. The GO biological process category was mainly enriched in regulation of peptidase activity, response to peptides, and endopeptidase activity. The GO cellular component category was mainly enriched in collagen-containing extracellular matrix, vesicle lumen, and cytoplasmic vesicle lumen. The molecular function category was mainly enriched in protein serine/threonine/tyrosine kinase, endopeptidase, and protein serine/threonine kinase activities (Figure 2B). DO upregulation was mainly enriched in musculoskeletal system cancer, connective tissue cancer, non-small cell lung carcinoma, bone cancer, female reproductive organ cancer, and breast carcinoma (Figures 2C,D). KEGG analysis revealed enrichment mainly in the pathways of neurodegeneration, multiple diseases, Alzheimer’s disease, PI3K-Akt signaling pathway, lipid and atherosclerosis, and Epstein-Barr virus infection (Figures 2E,F).

### 3.2 Target genes in glioblastoma

**FIGURE 3:**
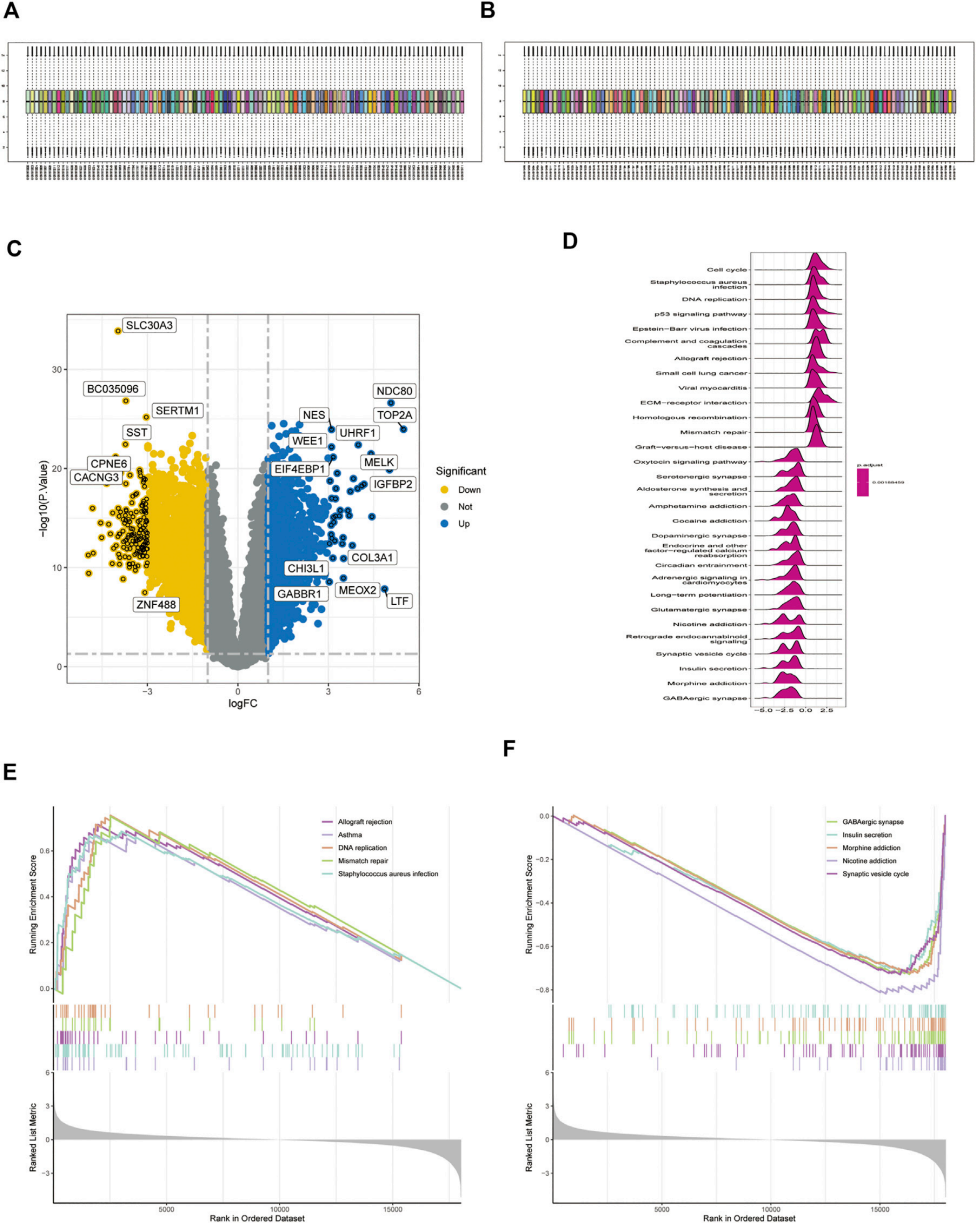
Expression of differentially expressed genes (DEGs) in the GSE4290 dataset. **(A,B)** Datasets were compared before and after normalization. **(C)** Volcano plot of DEGs in the GSE4290 dataset. **(D)** Ridge plots with normalized enrichment scores show the pathways where DEGs are most enriched in gene set enrichment analysis (GSEA). **(E,F)** GSEA analysis based on KEGG analysis.

Using normalization between arrays based on the GSE28424 dataset (Figures 3A,B), 3,135 genes were screened for differential expression between glioblastoma samples and normal tissue. Among the genes, 1,345 were upregulated and 1790 were downregulated (Supplementary Table S2); the top 20 genes are shown in a volcano plot (Figure 3C) and ridge plot (Figure 3D) drawn using R language for the glioblastoma group. Pathway enrichment was evaluated using GSEA pathway between the glioblastoma and control groups. The results showed that allograft rejection, asthma, DNA replication, mismatch repair, and *Staphylococcus aureus* infection activation were enriched in glioblastoma (Figure 3E). GABAergic synapses, insulin secretion, morphine addiction, nicotine addiction, and synaptic vesicle cycle were significantly inhibited (Figure 3F), suggesting that immune dysfunction plays an essential role in glioma development.

### 3.3 WGCNA

**FIGURE 4:**
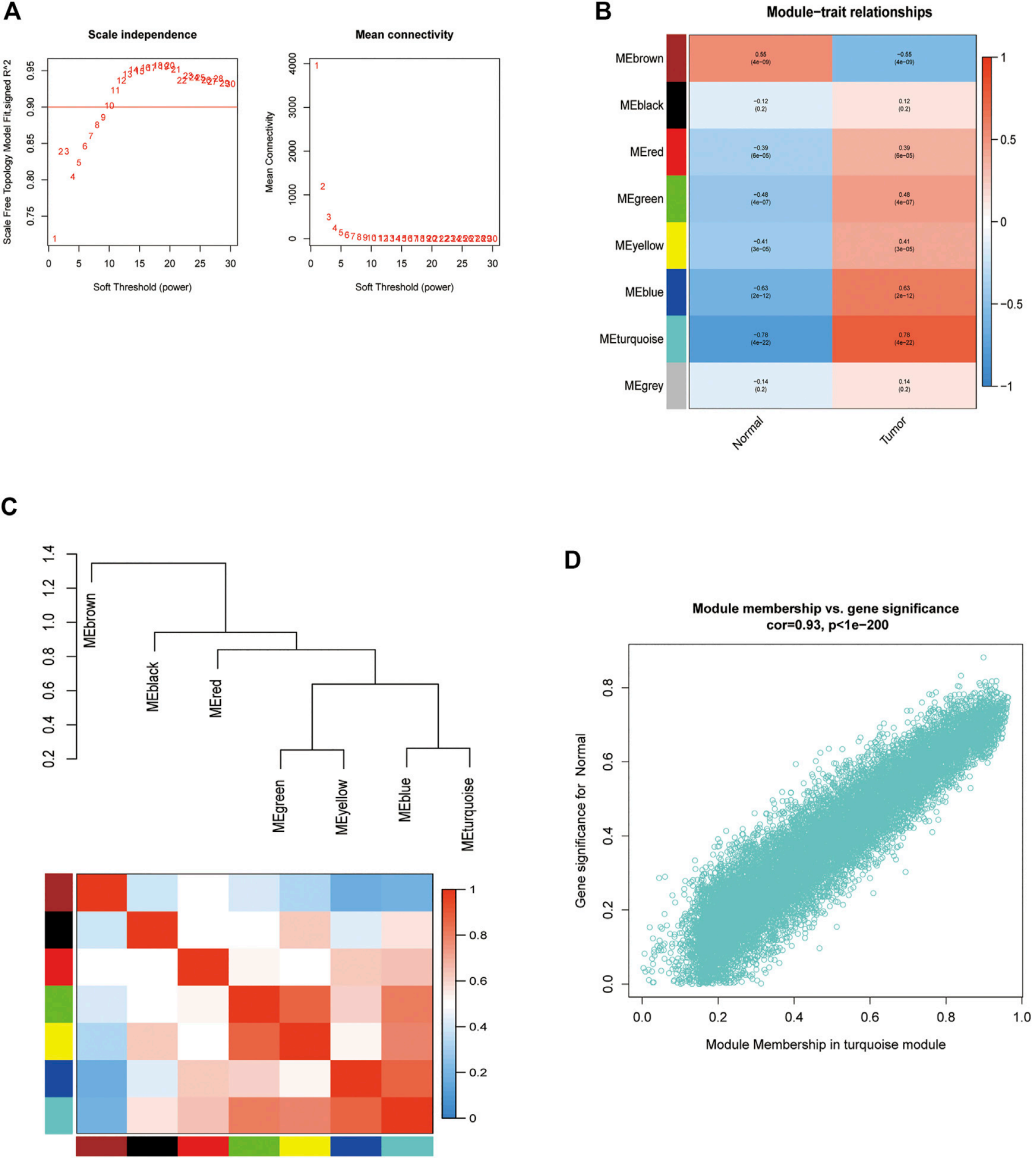
Enrichment levels in genomic weighted gene co-expression network analysis (WGCNA). **(A)**. Selection of soft thresholds. **(B,C)** Correlation of module eigengenes with glioblastoma. **(D)** Correlation of turquoise eigengenes with glioblastoma.

GSE4290 microarray data and clinical information were downloaded and pre-processed to obtain a final expression matrix of 100 samples corresponding to 23,323 genes. The 5,000 genes with the highest average expression were selected to create a gene co-expression module. The dataset was processed for outlier detection, which showed no significant outliers. Next, we directly analyzed the gene clustering module against the clinical grouping phenotypes. The soft threshold power was to 1–30, with *R* ^2^ > 0.9. The soft threshold power and mean connectivity were close to zero, indicating that the network was scale-free. Therefore, a soft threshold of 9 was chosen (Figure 4A). The topological overlap matrix and correlation matrix between genes were also computed. The topological overlap matrix was used to build a hierarchical clustering tree between genes, and merging of similar modules produced eight modules. The turquoise module showed the strongest correlation with glioblastoma (*r* = 0.78, P 0.01), as shown in Figures 4B,C. The scatter plot revealed a strong correlation between GS and MM within the turquoise module (correlation = 0.93, *p* < 0.01) (Figure 4D). Thus, the turquoise module may be a pivotal module linked to glioblastoma.

### 3.4 Functional enrichment analysis of genes within modules

**FIGURE 5:**
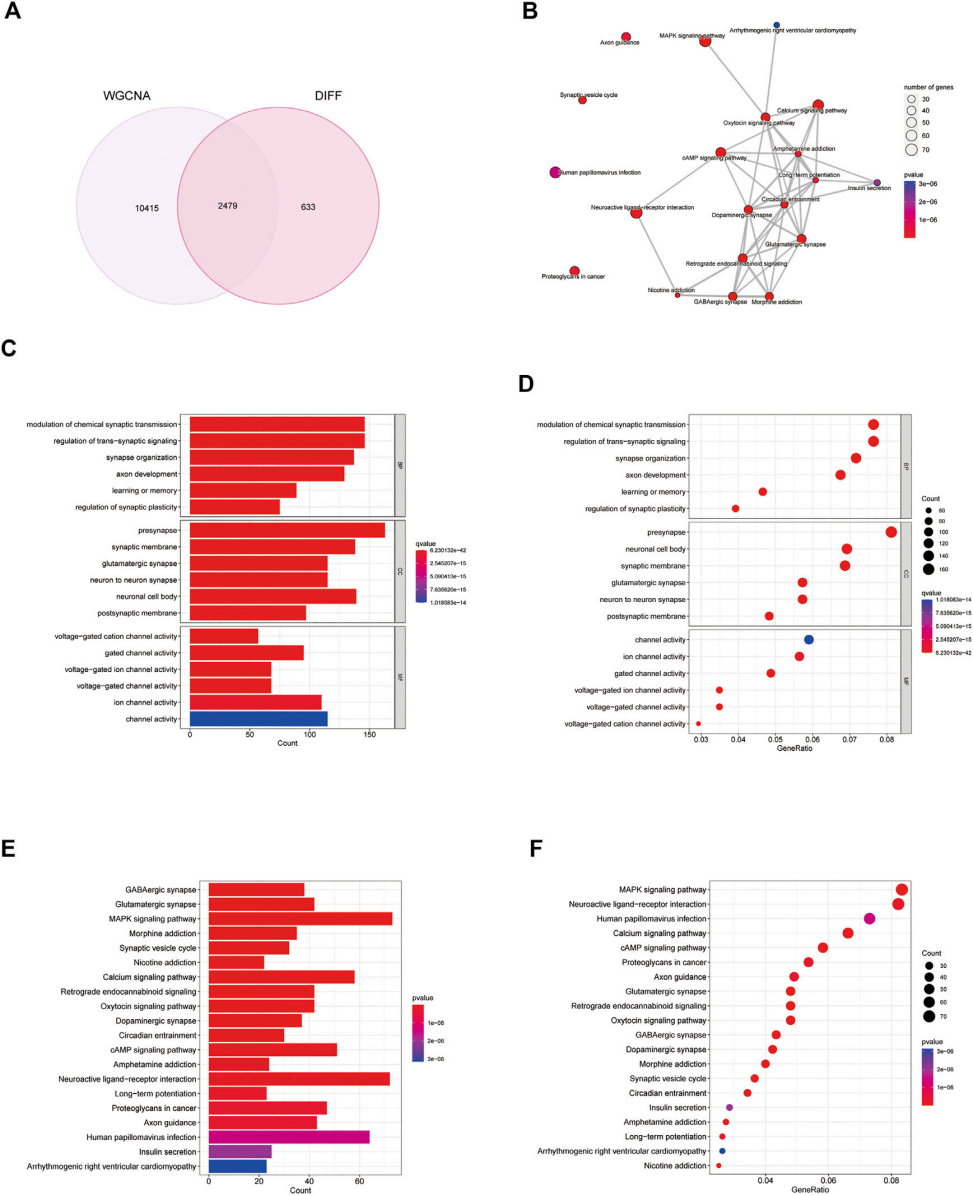
Analysis of differential genes and weighted gene co-expression network analysis (WGCNA) genes. **(A)** Venn diagram of differential genes and WGCNA genes. **(B–F)** Gene Ontology (GO) and Kyoto Encyclopedia of Genes and Genomes (KEGG) analysis of disease genes.

Genes in the turquoise module were compared with differential genes to identify disease-related genes in GO and KEGG analyses (Figure 5A). According to the GO enrichment results, the enriched pathways were mainly involved in modulation of chemical synaptic transmission, regulation of transsynaptic signaling, synapse organization, presynaptic membrane, and glutamatergic synapses. According to KEGG enrichment analysis, the enriched pathways were mainly involved in GABAergic synapses, glutamatergic synapses, MAPK signaling pathways, and morphine addiction (Figures 5B–F).

**FIGURE 6:**
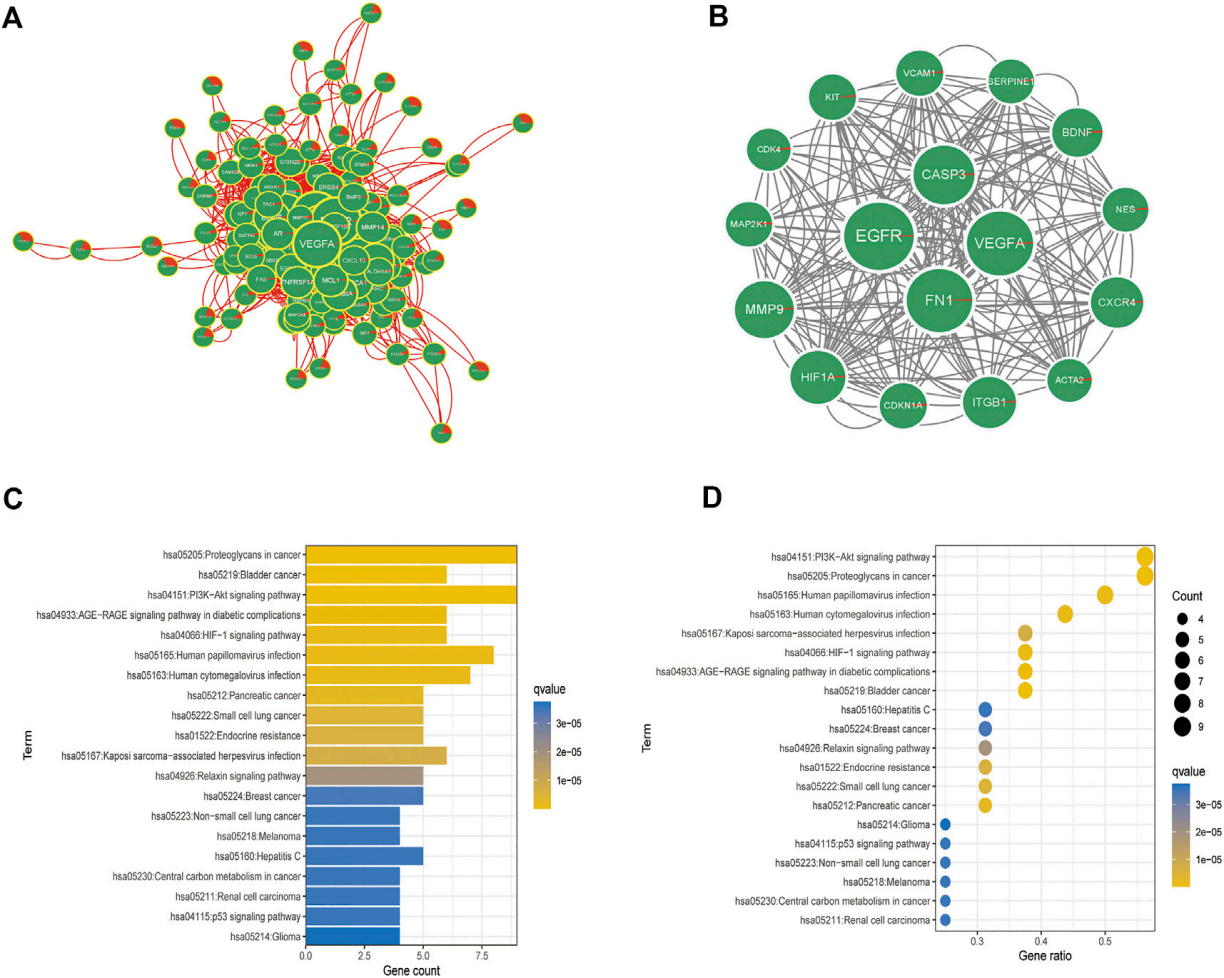
Analysis of crucial cluster genes. **(A,B)** Screening for essential subcluster genes. **(C,D)** Kyoto Encyclopedia of Genes and Genomes (KEGG) enrichment analysis of essential subcluster genes (top 10).

To construct the PPI network, 155 disease-related genes and molecular drug targets were imported into the STRING online database (version 11.0) (Figure 6A). Aberrant proteins were removed, resulting in a 154-protein interaction network. Cytoscape’s plugin code was used to identify 17 essential subpopulation genes (score = 13) (Figure 6B). Key cluster genes were upregulated for proteoglycans in cancer, bladder cancer, PI3K-Akt signaling pathway, AGE-RAGE signaling pathway in diabetic complications, HIF-1 signaling pathway, Kaposi sarcoma-associated herpesvirus infection, endocrine resistance, small cell lung cancer, pancreatic cancer, and human cytomegalovirus infection (Figures 6C,D). In addition to acting on cellular metabolic pathways, paclitaxel may be useful for diagnosing glioblastoma.

### 3.5 Differential expression of critical genes in tumor tissue and controls and prognostic analysis

**FIGURE 7:**
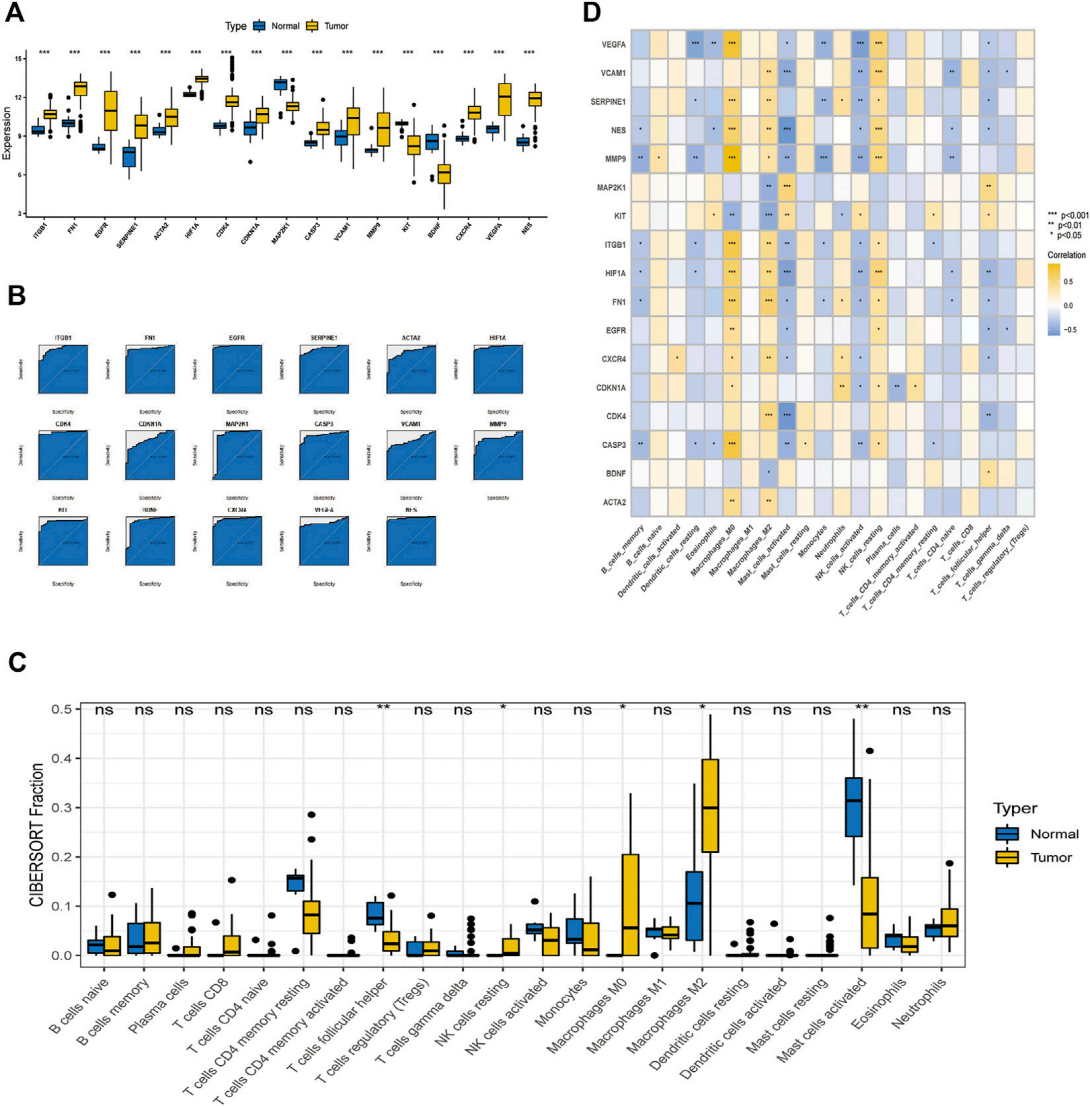
Relationship between crucial cluster genes and immune infiltration and receiver operating characteristic (ROC) prediction. **(A)** Differential expression of crucial subgroup genes in tumor and control tissues. **(B)** ROC curves of essential subcluster genes predicting disease onset. **(C)** Differential expression of immune function between tumor and control tissues. **(D)** Relationship between crucial cluster genes and immune infiltration.

As shown in the box plots, individual essential sub-cluster genes were significantly different between the disease and control groups (Figure 7A), and the ROC curves showed that all 17 essential sub-cluster genes had excellent robustness for glioblastoma (area under the ROC curve >0.6) (Figure 7B). The immune heat map showed that the significant regulatory targets of the critical cluster genes were mainly in the immune pathways of Macrophages_M0, Macrophages_M2, Mast_cells_activated, and T_cells_follicular_helper (Figures 7C,D), indicating that these genes are involved in regulating glioma immune function, which is consistent with previous studies (Wang et al., 2022).

### 3.6 Single-cell assay analysis

**FIGURE 8:**
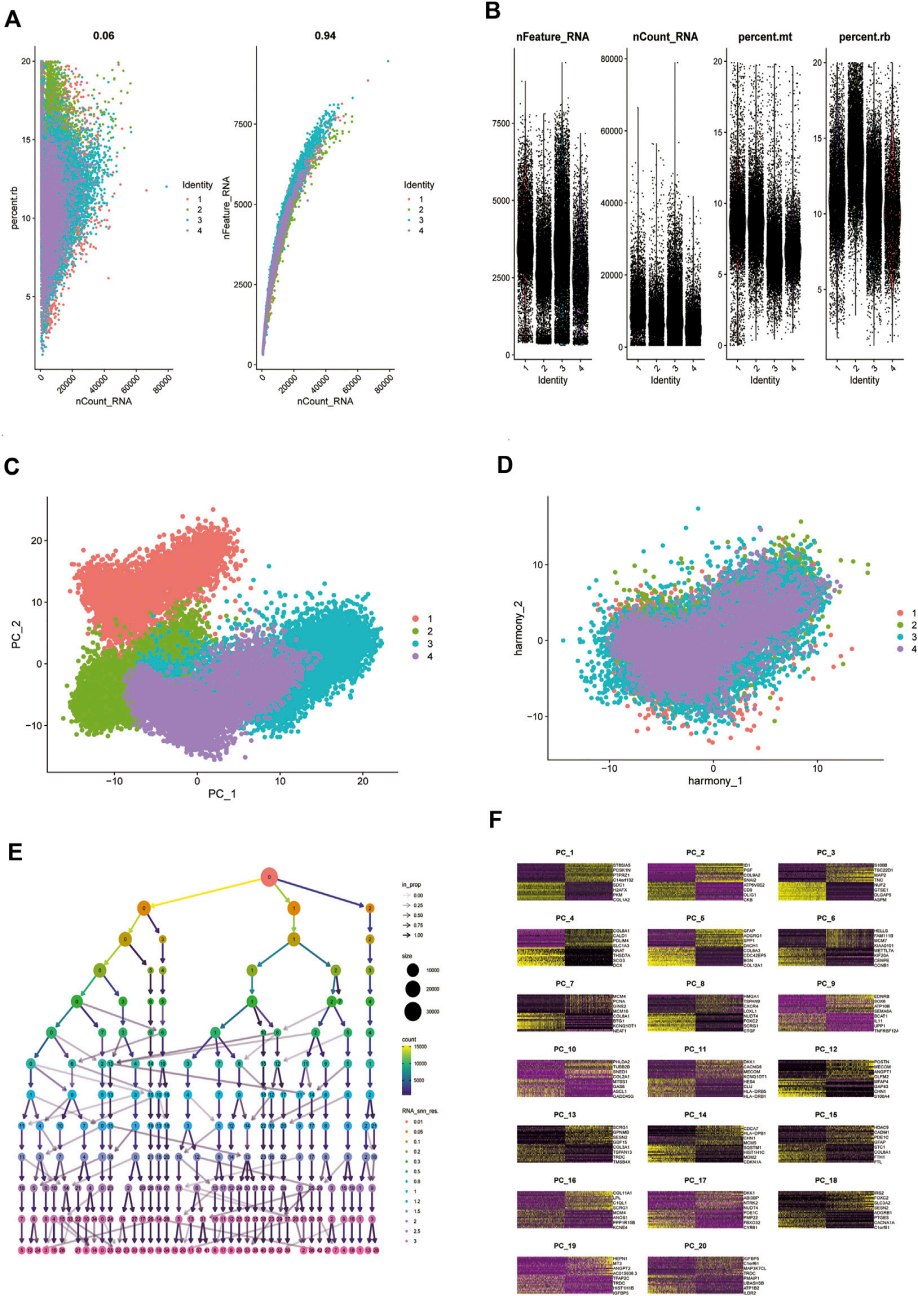
Comparison of single-cell analysis before and after normalization. **(A,B)** Quality control analysis of single-cell data sets. **(C,D)** Plots of principal component analysis (PCA) before and after standardization. **(E,F)** Resolution with principal components (PCs) to be confirmed.

**FIGURE 9:**
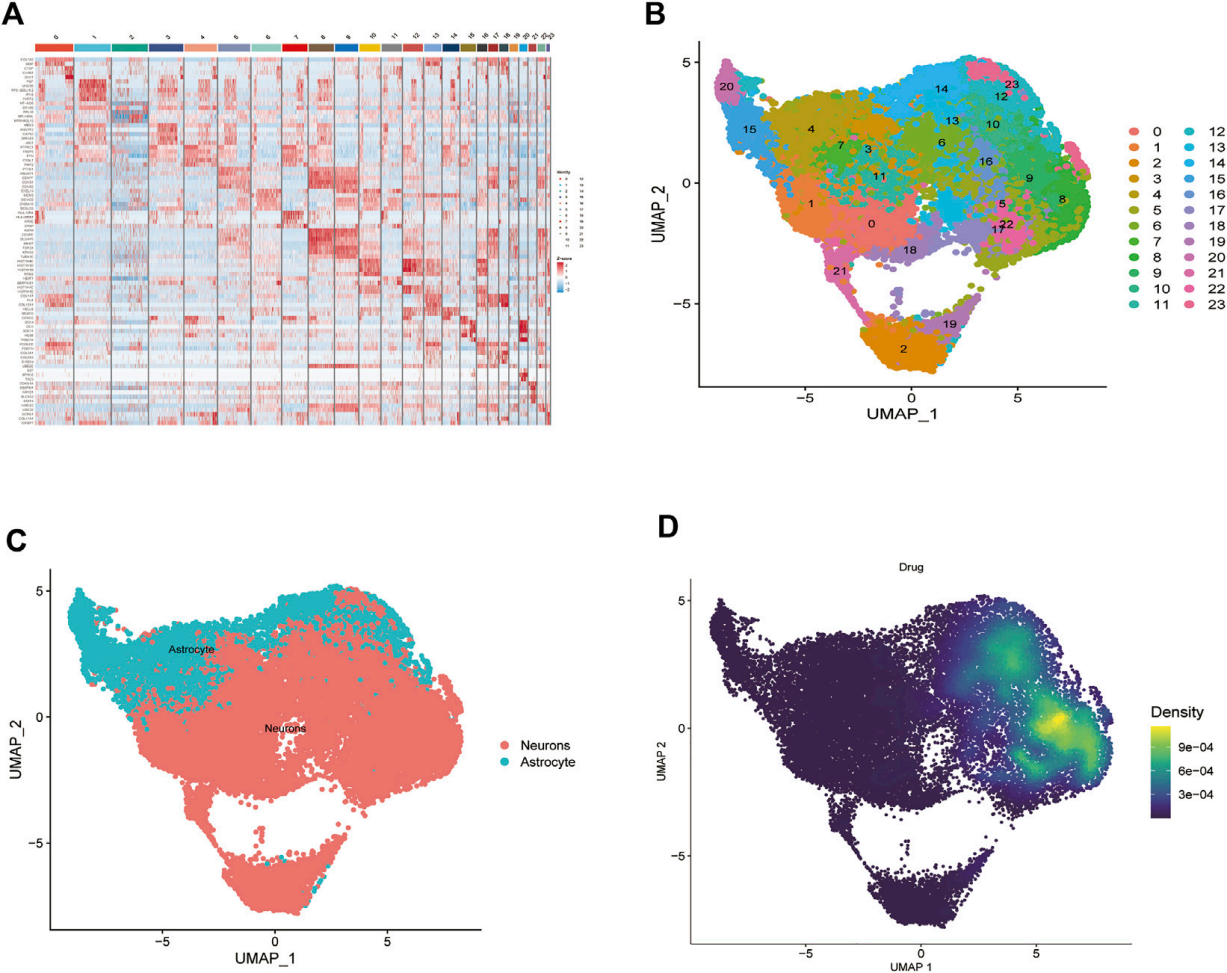
Paclitaxel pathways of action. **(A)** Heat map of each gene table level. **(B)** Unified manifold approximation and projection clustering into 24 clusters. **(C)** iTalk analysis identified two clusters. **(D)** Paclitaxel drug pathways of action.

Analysis of biopsy specimens from four patients with active glioblastoma showed a strong positive correlation between the measured gene expression and number of genes detected in the cells, both in normal and diseased tissues. In contrast, gene expression detected in the cells was not correlated with the percentage of mitochondria. Therefore, cells with >2,500 and <200 genes detected per cell and cells with a >5% mitochondrial percentage were filtered out to ensure the quality of the analyzed cells. Quality control and screening of single-cell sequencing of samples from patients with glioblastoma are shown in (Figures 8A,B). Principal component analysis plots were downscaled for cluster analysis (Figures 8C,D); the cluster tree was scaled to a resolution of 1.5 (Figure 8E), and the principal component value was 16 (Figure 8F). The heatmap shows each gene type (Figure 9A). Unified manifold approximation and projection showed 24 cell clusters (Figure 9B), with different categories of cells labeled with different colors. Relevant genes were retrieved using the Cellmaker database and intersected with the gene corresponding to each unified manifold approximation and projection cluster (Figure 9C). The cellular distribution of drug target AUCell functional scores showed that paclitaxel acts mainly on neuronal cells (Figure 9D).

## 4 Discussion

Glioblastoma is a highly malignant primary malignant tumor; and WHO grade III and IV malignant glioblastoma is a common type of high-grade glioblastoma (Mitobe et al., 2022; Yilmaz et al., 2022), with no apparent boundary between the tumor tissue and surrounding tissue. Therefore, the efficacy of surgery alone in treating malignant glioblastoma is poor and results in a median survival of only approximately 10 months (Liu et al., 2017b). Paclitaxel is a traditional anti-tumor drug effective against ovarian cancer, colorectal cancer, breast cancer, and glioblastoma (Dorsey et al., 2009). In recent years, more and more studies have shown the therapeutic effect of paclitaxel on glioma, and it has been verified *in vivo* and *in vitro* experiments (Xie et al., 2006; Jiang et al., 2011b). Substantial progress has been made in the *in vitro* treatment of glioma with continuous delivery of paclitaxel through biodegradable materials (Xie and Wang, 2006). In addition, it has been shown that the treatment of glioblastoma with tumor-targeted gene vectors and brain-targeted micelles and paclitaxel co-delivery has achieved good efficacy in mouse experiments (Zhan et al., 2012). In this experiment, we used single-cell sequencing data for in-depth mining, and advances in this next-generation sequencing approach have enabled genomic analysis of single cells, which is beneficial to reveal heterogeneous tumors and has an important role in the treatment of cancer (Navin and Hicks, 2011; Zhang et al., 2016). In addition, single-cell sequencing is likely to improve several aspects of pharmacology, including precise targeting of drugs, cellular receptors, and deeper mechanisms of action (Lee et al., 2014; Heath et al., 2016).

We identified 1,010 target genes related to paclitaxel using the SwissTargetPrediction, CTD, BindingDB, and TargetNet databases. GO analysis showed enrichment in the regulation of peptidase activity, response to peptide, and regulation of endopeptidase activity in the biological process category. In the cell component category, enrichment was observed in collagen-containing extracellular matrix, vesicle lumen, and cytoplasmic vesicle lumen. For molecular function, we observed enrichment in protein serine/threonine/tyrosine kinase activity, endopeptidase activity, and protein serine/threonine kinase activity (Figure 2B). DO enrichment analyses were mainly enriched in musculoskeletal system cancer, connective tissue cancer, non-small cell lung carcinoma, bone cancer, female reproductive organ cancer, and breast cancer; pathways related to neurodegeneration-multiple diseases, Alzheimer disease, PI3K-Akt signaling pathway, lipid and atherosclerosis, Epstein-Barr virus infection were enriched according to KEGG analysis. These results suggest that paclitaxel can treat a variety of tumor cells by regulating the body’s peptidase activity and other signaling pathways. The 3,135 genes differentially expressed between glioblastoma samples and normal tissues, including 1,345 upregulated genes and 1790 downregulated genes, were enriched in allograft rejection, asthma, DNA replication, mismatch repair, and glioblastoma. Replication, mismatch repair, and *S. aureus* infection pathway were activated. GABAergic synapse, insulin secretion, morphine addiction, nicotine addiction, and synaptic vesicle cycle pathways were significantly inhibited, suggesting that immune function was overactivated in the tumor tissues (Perelroizen et al., 2022).

We also identified 2,479 key genes involved in disease progression. Fifty-three key subgroups of genes were enriched in proteoglycans in cancer, bladder cancer, and PI3K-Akt signaling pathways. Cybersport analysis was used to calculate the number of genes in 22 immune cells. The results showed that the drug-disease critical cluster of genes was primarily targeted by one of the major immune pathways and other pathways to exert a therapeutic effect. Single-cell data analysis showed that the main target of paclitaxel was neuronal cells, which is consistent with previous results (Duhamel et al., 2018).

Our results suggest that paclitaxel improves the prognosis of glioblastoma by acting on neuronal cells and modulating immunity (Xue et al., 2017). We also identified *ITGB1*, *FN1*, *EGFR*, *SERPINE1*, *ACTA2*, *HIF1A*, *CDK4*, *CDKN1A*, *MAP2K1*, *CASP3*, *VCAM1*, *MMP9*, *KIT*, *BDNF*, *CXCR4*, *VEGFA*, and *NES* as essential cluster genes, which agrees with the results of previous related studies. Li et al. suggested that GLIPR1 enhances the proliferation, migration, and invasion of glioblastoma and may be involved in activation of the TIMP1-CD63-ITGB1-AKT signaling pathway, which is a potential target for the clinical prevention or management of glioblastoma (Jiang et al., 2022). Wu et al. (2021) found that the regulation of ITGB1 expression promotes progression, suggesting an essential role for ITGB1 in glioblastoma. Andersson et al. (2010) demonstrated that regulation of the EGFR pathway is involved in glioblastoma progression and that specific genotypes of the EGFR gene may be associated with glioblastoma risk. According to Ohtaki et al. (2017), ACTC1 is as an independent prognostic and aggressive marker of gliomas. In addition, Wu et al. (2022) demonstrated that CXCR4 promotes the proliferation of GICs through the KLF5/BCL2L12-dependent pathway. These essential cluster genes may have good predictive efficacy for glioblastoma and are essential for glioblastoma development. Further studies are needed to identify the molecular mechanisms involved in the immune response to glioblastoma.

Our experiments still have some limitations, lack prospective cohort and *in vitro* experiments, and the specific molecular mechanism of paclitaxel affecting glioma remains unclear, but our advantage lies in the clear description of paclitaxel target cells based on single-cell data, which makes an important contribution to the study of the specific molecular mechanism in the next step.

## 5 Conclusion

We examined the interactions and molecular mechanisms of paclitaxel in glioblastoma. Paclitaxel and glioblastoma synergistically affected differentially regulated genes. We used modern network medicinal theories to investigate the molecular biological mechanisms of paclitaxel in glioblastoma, which may help guide clinical practice. In future studies, we will validate these results in pharmacological and molecular biology experiments.

## Data Availability

The original contributions presented in the study are included in the article/Supplementary Material, further inquiries can be directed to the corresponding author.
